# An elevational gradient in floral traits and pollinator assemblages in the Neotropical species *Costus guanaiensis* var. *tarmicus* in Peru

**DOI:** 10.1002/ece3.10314

**Published:** 2023-07-28

**Authors:** Rossana Maguiña‐Conde, Dorali Zuñiga‐Rivas, Kathleen M. Kay

**Affiliations:** ^1^ Ecology and Evolutionary Biology Department University of California Santa Cruz Santa Cruz California USA; ^2^ Laboratorio de Entomología Universidad Nacional San Antonio Abad del Cusco Cusco Peru

**Keywords:** Euglossini, floral adaptation, hummingbird, orchid bee, Phaethornithinae, plant‐pollinator interaction

## Abstract

Different populations of plant species can adapt to their local pollinators and diverge in floral traits accordingly. Floral traits are subject to pollinator‐driven natural selection to enhance plant reproductive success. Studies on temperate plant systems have shown pollinator‐driven selection results in floral trait variation along elevational gradients, but studies in tropical systems are lacking. We analyzed floral traits and pollinator assemblages in the Neotropical bee‐pollinated taxon *Costus guanaiensis* var. *tarmicus* across four sites along a steep elevational gradient in Peru. We found variations in floral traits of size, color, and reward, and in the pollinator assemblage along the elevational gradient. We examined our results considering two hypotheses, (1) local adaptation to different bee assemblages, and (2) the early stages of an evolutionary shift to a new pollinator functional group (hummingbirds). We found some evidence consistent with the adaptation of *C. guanaiensis* var. *tarmicus* to the local bee fauna along the studied elevational gradient. Corolla width across sites was associated with bee thorax width of the local most frequent pollinator. However, we could not rule out the possibility of the beginning of a bee‐to‐hummingbird pollination shift in the highest‐studied site. Our study is one of the few geographic‐scale analyses of floral trait and pollinator assemblage variation in tropical plant species. Our results broaden our understanding of plant‐pollinator interactions beyond temperate systems by showing substantial intraspecific divergence in both floral traits and pollinator assemblages across geographic space in a tropical plant species.

## Graphical abstract

We analyzed floral traits and pollinator assemblages in the Neotropical species *Costus guanaiensis* var. *tarmicus* along a steep elevational gradient in Peru. Results show floral traits and pollinator assemblage variation along the elevational gradient. We examined our results considering two hypotheses: (1) local adaptation to different bee assemblages and (2) the early stages of an evolutionary shift to a new pollinator functional group (hummingbirds).

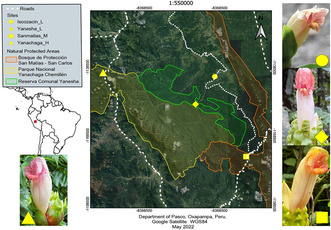

## Resumen

Diferentes poblaciones de una especie de planta pueden adaptarse a sus polinizadores locales y, en consecuencia, divergir en los rasgos florales. Los rasgos florales están sujetos a la selección natural impulsada por los polinizadores para mejorar el éxito reproductivo de la planta. Los estudios en sistemas de plantas de zonas templadas han demostrado que la selección impulsada por los polinizadores da como resultado una variación de los rasgos florales a lo largo de gradientes de altitud, pero faltan estudios en sistemas de plantas de zonas tropicales. Analizamos las características florales y los ensamblajes de polinizadores en el taxón Neotropical polinizado por abejas *Costus guanaiensis* var. *tarmicus* en cuatro sitios a lo largo de un empinado gradiente altitudinal en Perú. Encontramos variación en los rasgos florales de tamaño, color y recompensa, y en los ensamblajes de polinizadores a lo largo del gradiente altitudinal. Examinamos nuestros resultados considerando dos hipótesis, (1) la adaptación local a diferentes ensamblajes de abejas polinizadoras y (2) las primeras etapas de un desplazamiento evolutivo a un nuevo grupo funcional polinizador (colibríes). Encontramos evidencia que respalda la adaptación de *C. guanaiensis* var. *tarmicus* a la fauna de abejas local a lo largo del gradiente altitudinal estudiado. El ancho de la corola de la flor a lo largo de los sitios de estudio se asoció con el ancho del tórax de la especie de abeja local más frecuentemente registrada en las flores. Sin embargo, no pudimos descartar la posibilidad del comienzo de un desplazamiento de polinización de abeja a colibrí en el sitio más alto estudiado. Nuestro estudio es uno de los pocos análisis a escala geográfica de variación de rasgos florales y ensamblaje de polinizadores en una especie tropical. Nuestros resultados amplían la comprensión de las interacciones planta‐polinizador más allá de los sistemas templados al mostrar una sustancial divergencia intraespecífica tanto en los rasgos florales como en los ensamblajes de polinizadores en una especie tropical.

## 1 INTRODUCTION

Floral traits are evolutionarily labile and subject to pollinator‐driven natural selection (Armbruster, 1985, 1993; Carr & Fenster, 1994). Selection often changes floral traits in a way that enhances plant reproduction by increasing attraction and reward traits for pollinators, resulting in higher visitation rates, and by shaping traits in a way that improves the pollinator‐flower fit, resulting in higher pollination efficiency (Stebbins, 1970). Thus, different plant populations of the same species adapt to local pollinators and diverge in floral traits accordingly (Anderson et al., 2010; Anderson & Johnson, 2008, 2009; Galen, 1996; Johnson & Steiner, 1997; Maad et al., 2013; Medel et al., 2007; Nattero et al., 2011; Newman et al., 2015; Thompson, 2005). Understanding floral adaptation to pollinators can provide insight into major evolutionary processes involved in angiosperm diversification, such as pollination shifts (Kay & Sargent, 2009).

Different populations of plants occurring along elevational gradients can exhibit floral phenotypic variation mediated by pollinator selection (Galen, 1996; Maad et al., 2013; Nattero et al., 2011; Zhao & Wang, 2015). Studies of multiple bee‐pollinated species found an increase in flower size with elevation (Galen, 1996; Maad et al., 2013; Malo & Baonza, 2002). Similarly, bee community composition can change with elevation, resulting in an increase in the mean body size in the community (Hoiss et al., 2012; Malo & Baonza, 2002). Research on the association between floral traits and bees' pollinator assemblages concluded that selection drives the increase of flower size along elevational gradients to improve the fit of bigger pollinators with the floral reproductive structures (Galen, 1996; Maad et al., 2013). Changes in pollinator assemblages can result in rapid floral trait divergence over a single generation (Galen, 1996) or even from one year to the next one (Schemske & Horvitz, 1989). To date, however, few studies have focused on floral traits and pollinator assemblages variation within species along elevational gradients and even fewer studies have been conducted in tropical areas (but see Dellinger et al., 2021 for a study focused on bee‐to‐hummingbird pollination shifts; and Klomberg et al., 2022 and Nattero et al., 2011 for studies on geographic floral trait variation without pollination shifts). It is imperative to devote efforts to existing research programs on tropical species, such as species in the Neotropical spiral gingers (genus *Costus*, reviewed in Moreira‐Hernández & Muchhala, 2019, and Thomson & Wilson, 2008), for a comprehensive understanding of pollination shifts across temperate and tropical systems (Thomson & Wilson, 2008).

Bee diversity and abundance decrease with elevation in both temperate and tropical areas, (Arroyo et al., 1982; Hoiss et al., 2012). In contrast, vertebrate pollinators, such as hummingbirds, can inhabit a wider elevational range than bees and perform well as pollinators. For instance, in rainy conditions at high elevations in Mexico, bird‐pollinated plants were more effectively pollinated than closely related bee‐pollinated plants (Cruden, 1972). The abundance and diversity of hummingbirds are higher in tropical versus temperate areas (Greenwalt, 1960). Thus, it is more likely that in the tropics, pollinator assemblages at high‐elevation sites might include the presence of hummingbirds as pollinators, in addition to larger bee pollinators. There is however little empirical evidence regarding environmental factors, such as those associated with elevation (Thomson & Wilson, 2008), driving specific changes in pollinator assemblage from bees to hummingbirds (but see Dellinger et al., 2021), or from bees to a mixed pollinator assemblage.

*Costus* (Costaceae), a species‐rich genus of herbaceous plants, has several Neotropical species pollinated either by orchid bees (Apidae: Euglossini) or hermit hummingbirds (Phaethornithinae, Kay & Schemske, 2003). Bee‐ and hummingbird‐pollinated species have different suites of floral traits (Kay & Grossenbacher, 2022). Bee‐pollinated species generally have long, wide, and pale flowers, and a large petaloid labellum colored with two thick yellow lines that presumably act as nectar guides. In contrast, hummingbird‐pollinated species have short, narrow, and brightly colored flowers with a reduced labellum (Kay & Grossenbacher, 2022). Flowers of *Costus guanaiensis* var. *tarmicus* seem to differ in visual floral traits among sites distributed along an elevational gradient from the Amazonian basin to the Andes mountains in Peru (up to 2000 meters above sea level [m a.s.l.]). These observations and the broad elevational distribution of *C. guanaiensis* var. *tarmicus* offer a natural setup to study floral divergence across a tropical elevational gradient. We addressed the following questions: (1) Do floral traits vary among sites along an elevational gradient? (2) Do pollinator assemblages vary among sites along an elevational gradient? and (3) Is the pollinator assemblage variation associated with floral trait variation? To answer these questions, we conducted an analysis of *C. guanaiensis* var. *tarmicus* floral traits (size, color, and reward) and its pollinator assemblages at four sites along a 1000 m elevational gradient in Peru.

We had two hypotheses about how floral traits and pollinator assemblages of *C. guanaiensis* var. *tarmicus* might vary with elevation. First, if *C. guanaiensis* var. *tarmicus* is adapted to pollination by the local bee fauna (local bee adaptation hypothesis), we expected that the floral traits and pollinator assemblages would covary among sites. Given that there may be fewer bees in the highest site, and thus, a lower bee visitation rate compared to lower sites, attraction traits might be exaggerated there; for instance, the yellow lines (nectar guides) in the labellum would cover a wider area and the nectar sugar concentration would be greater at the high site compared to lower sites. Pollinator assemblages at all sites would comprise orchid bees, but the highest elevation site might receive visits from bigger bee species than those at lower elevation sites (Bishop & Armbruster, 1999; Galen, 1996; Maad et al., 2013). If so, we expect that the highest elevation site would have bigger flowers than lower elevation sites.

Alternatively, if in the high elevation site, *C. guanaiensis* var. *tarmicus* is not only adapted to the local bee fauna but also to hummingbird pollination (pollinator shift hypothesis), we expect that the floral traits would vary with elevation, as we explained in the previous hypothesis, but that the floral traits at the highest site would include traits that deter bees and attract or fit hummingbirds. Thus, we would not detect a flower and bee morphology correlation along the elevational gradient. For example, for a better hummingbird‐flower fit, the flowers at the highest site would be the shortest to allow hummingbirds to reach the nectar and the narrowest to improve pollen transfer. To deter the bees, the flowers at the highest site would have thinner or absent yellow lines (nectar guides) on the labellum. In addition, the nectar sugar concentration would be the lowest at the highest site, matching similar levels to known hummingbird flower sources (Baker, 1975; Bolten & Feinsinger, 1978). A dilute nectar would deter bees by reducing their sugar intake rate (Cnaani et al., 2006; Harder, 1986; Heinrich, 1975). Pollinator assemblages at the highest site would comprise orchid bees and hummingbirds, but with the lowest bee visitation rate and the highest hummingbird visitation rate compared to lower sites.

## 2 MATERIALS AND METHODS

### 2.1 Study system and sites

Costaceae is a family of monocots native to tropical climates of Central America, South America, Asia, and Africa (Maas, 1972). The studied species belongs to the Neotropical *Costus* clade with species distributed from Mexico to Brazil, encompassing the Andes mountainous regions, with species present in low‐ to mid‐elevation (0–2000 m a.s.l.; Maas, 1972; Vargas et al., 2020). *Costus* typically produce a single nectar‐rich flower per plant per day, and pollinators are thought to travel on traplines between widely spaced plants. Bee pollination is ancestral in the Neotropical clade, but there have been at least 11 independent shifts to hummingbird pollination (Kay & Grossenbacher, 2022; Vargas et al., 2020), motivating the hypothesis that geographic divergence in floral traits might be associated with adaptation to hummingbirds.

*Costus guanaiensis* is a polyphyletic species with multiple named varieties (Maas, 1972; Vargas et al., 2020). Here we focus solely on the taxon *C. guanaiensis* var. *tarmicus. Costus guanaiensis* var. *tarmicus* ranges from 250 to 2000 m a.s.l. in the Peruvian Amazon and Andes (www.tropicos.org). There are no previous studies on the pollination biology of this specific taxon, although there are records of orchid bees pollinating *C. guanaiensis* var. *macrostobilus* (Schemske, 1981; Sytsma & Pippen, 1985). The common name of these plants in Central and South America is “caña agria,” due to their resemblance with sugar cane plants (Maas, 1972).

**FIGURE 1:**
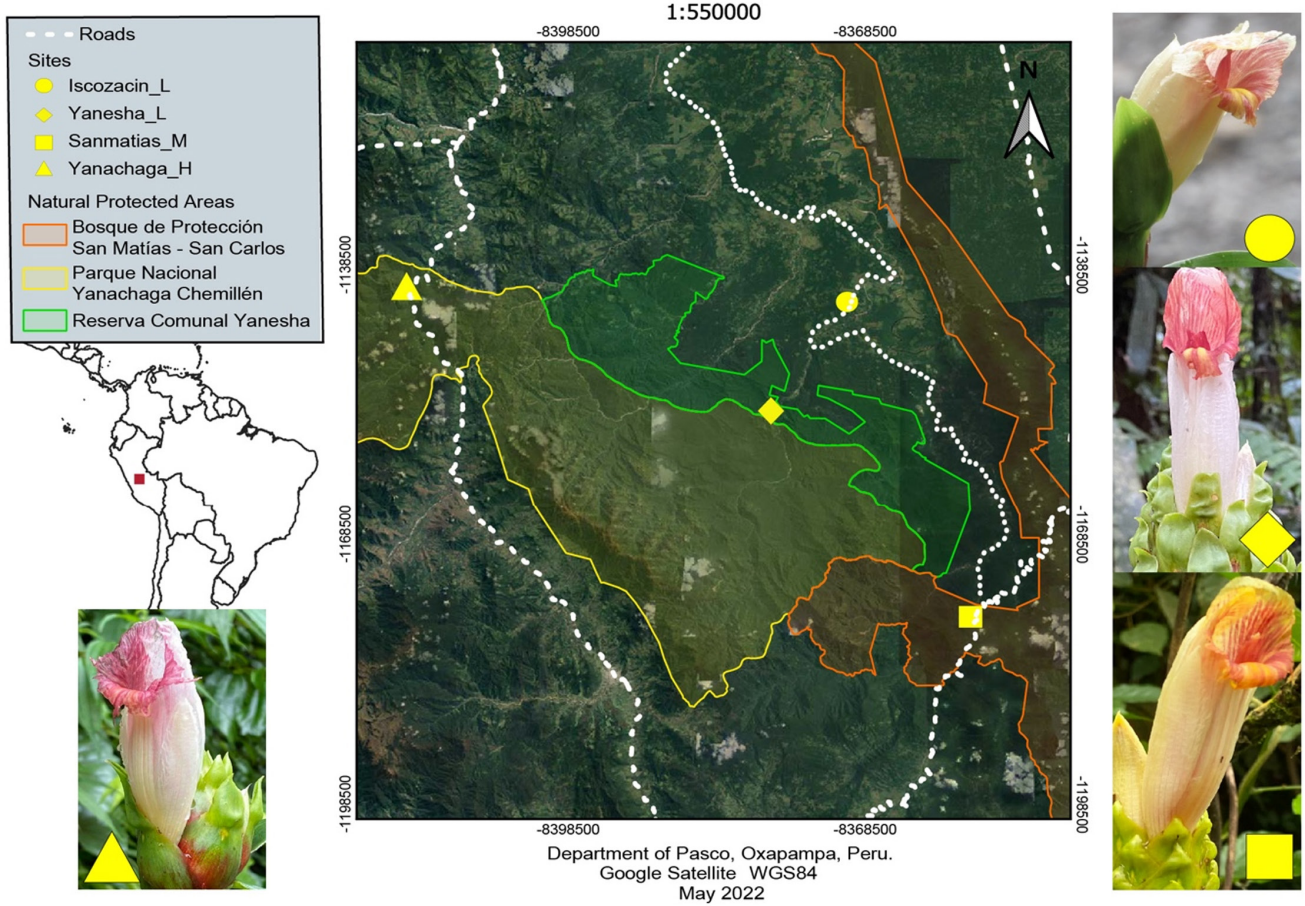
Location of our sampling sites in Pasco‐Peru, Iscozacin_L between 280 and 350 m a.s.l. (meters above sea level), Yanesha_L between 300 and 400 m a.s.l., Sanmatias_M between 500 and 700 m a.s.l., and Yanachaga_H between 1000 and 1200 m a.s.l. Flower picture from each sampling site (by R. Maguiña‐Conde). Map elaborated by E. Morales.

We reviewed herbaria collections (CUZ, HOXA, HUNMSM, MOBOT) of *C. guanaiensis* to locate populations of *C. guanaiensis* var. *tarmicus* in different elevations, in areas of relatively easy access by terrestrial routes and close to research stations or towns. We sampled *C. guanaiensis* var. *tarmicus* at four sites during its flowering season (November–February, rainy season in the Amazon) in 2019–2020 and 2020–2021. The sites are in the following areas and elevations: (1) surroundings of Iscozacin town between 280 and 350 m a.s.l. (low elevation site, hereafter Iscozacin_L), (2) Reserva Comunal Yanesha between 300 and 400 m a.s.l. (low elevation site, hereafter Yanesha_L), (3) Bosque de Protección San Matías San Carlos between 500 and 700 m a.s.l. (middle elevation site, hereafter Sanmatias_M), (4) Parque Nacional Yanachaga Chemillén between 1000 and 1200 m a.s.l. (high elevation site, hereafter Yanachaga_H). During the 2019–2020 sampling season, we worked at sites Yanesha_L, Sanmatias_M, and Yanachaga_H but concluded that the logistics of working at Yanesha_L were too complicated to go back to the following sampling season. Then, we decided to sample the Iscozacin_L site during 2020–2021 because we found a population of *C. guanaiensis* var. *tarmicus* there (not reported in the herbaria) more easily accessible than Yanesha_L. For the analysis, we decided to keep all four sites due to habitat conservation differences between the two low sites. All the sites are in the Department of Pasco on the eastern side of the Andes and western Amazonia in central Peru (Figure 1).

### 2.2 Floral phenotype analysis

We measured flower size, color corresponding to the nectar guides area, and nectar sugar concentration to quantify variation in floral traits among sites. We collected 9–11 flowers at each site to analyze flower size. In the morning, we searched for fresh flowers, removed them from the plants, and transported them in 50 mL thick plastic tubes to the research station/hotel, where we had a photography station set up. If any flower withered or damaged during the transportation, we did not use it for photos and waited to collect new flowers the next day. We took pictures of the flowers standing in molding clay to achieve a straight axis parallel to the camera. We took pictures of the frontal, lateral, and internal faces of each flower next to a ruler. From the pictures, we measured (in mm) 12 different floral traits using ImageJ (Rasband, 1997–2018). From the frontal view, we measured labellum length, labellum width, and corolla width. From the lateral view, we measured labellum length, corolla length, and corolla width. From the internal view, we measured petaloid stamen length (measured above the stigma), internal and horizontal labellum width, internal and diagonal labellum width, the distance between the anthers and the labellum, the distance between the anthers and the corolla base, and corolla tube length (see Appendix 1 for floral traits reference).

We performed a Principal Components Analysis (PCA) on floral traits measurements using the *prcomp* function in R (R Core Team, 2020). To assess if the floral traits differ among the sites, we applied a PERMANOVA followed by multiple pairwise comparison tests using the functions *adonis* (Thioulouse et al., 2018) and *pairwise.adonis* (Martinez Arbizu, 2020) from the package “vegan,” respectively. Also, we assessed how the flowers vary specifically in length and width. To compare the floral length, we applied ANOVA tests to the frontal and lateral measurements of labellum length and the lateral measurement of corolla length. For the floral width, we applied ANOVA tests to the frontal and lateral measurements of the corolla width. Following ANOVAs, we analyzed pairwise comparisons using the post hoc test function *TukeyHSD*.

To quantify the colors corresponding to the nectar guides area, we took pictures of five flowers from Iscozacin_L, Sanmatias_M, and Yanachaga_H sites in 2021. We could not take pictures of Yanesha_L flowers due to the site's inaccessibility during our second sampling season. We took pictures of the top part of the floral labellum. We placed the flower next to a Spyderckr color card that served as the gray standard. We used a Sony Alpha R7 full spectrum converted camera, a UV transmission lens model Nikon EL‐Nikkor 80mm f5.6, a visible‐spectrum‐pass filter (UV/IR Cut Hot Mirror 39 mm lens filter, Kolari Vision, US), and a UV‐pass filter (UV Bandpass 39 mm lens filter, Kolari Vision, US). We took pictures under natural daylight between 1000 and 1200 h, avoiding moments when clouds were covering direct sunlight. We took several pictures with each filter varying the exposure levels to select the best shot for the analysis. We assessed the nectar guides area on the labellum using the MicaToolbox plugin (Troscianko & Stevens, 2015) in ImageJ.

First, we generated a multispectral image using one flower picture taken with the visible‐spectrum‐pass filter and one taken with the UV‐pass filter. Then, we converted the multispectral image to a honeybee cone catch image (using the cone catch model available in the MicaToolbox software). Next, we acquired a presentation image for human vision from which we can distinguish colors seen by bees but not humans. These colors include the UV reflectance ones that we assumed corresponded to the flower's nectar guides. In the image, they appeared in dark pink‐purple colors. We converted this image to RGB colors and saved it as a jpeg image. Then, we converted the jpeg image to an 8‐bit color type with 256 colors (maximum number available). By doing so, we aimed to better define the different color areas, especially those with dark pink‐purple colors. Next, we converted the generated image to an 8‐bit grayscale type to measure the dark pink‐purple nectar guides area using the threshold settings. Then, we adjusted the threshold levels to identify the pixels corresponding to the nectar guides area. Finally, we used the measure command to get the nectar guides fraction area on the labellum (as the percentage area occupied on the labellum, see Appendix 2 for examples of the image processing). We compared the nectar guides fraction area among sites with a Kruskal–Wallis test, followed by a post hoc Dunn's test (due to the small sample size) for pairwise comparisons.

To quantify nectar sugar concentration, we collected nectar from 10 to 13 fresh flowers per site, between 0700 and 1030 h, using a capillary tube. The flowers we used were bagged the previous afternoon to avoid floral visitors taking the nectar before us. We placed a drop of the nectar in a refractometer (Eclipse refractometer, Bellingham + Stanley, UK) to record the sugar concentration in degrees Brix. We compared nectar sugar concentration among sites with an ANOVA test followed by Tukey's post hoc comparisons.

### 2.3 Pollinator assemblage analysis

We recorded pollinators visiting the flowers of *C. guanaiensis* var. *tarmicus*. We placed cameras with motion detection systems next to the inflorescence for 1–10 h per day. We used Canon Powershot SX530 cameras with the Canon Hack Development Kit (CHDK Development Team, 2018) installed in the SD card to enable the motion detection feature in the camera. The number of inflorescences and hours recorded varied among sites (Table 1) depending on the number of plants with flowers available and the number of days that we worked at each site. Our sample unit was each plant‐day combination. We recorded pollinator activity for up to 21 days at Iscozacin_L, Sanmatias_M, and Yanachaga_H. At Yanesha_L, we only recorded the flowers for 4 days due to site inaccessibility in our second sampling season. We watched the videos to record floral visitors and identify those that contacted reproductive organs (legitimate visits), thus acting as pollinators.

**TABLE 1:** Pollinator counts and visitation rate per hour to inflorescences of four populations of *Costus guanaiensis* var. *tarmicus* along an elevational gradient.

Site	# plants	# Inflores‐cences	# hours recorded	Total bee visits	Total hummingbird visits	Mean bee visitation rate	Mean hummingbird visitation rate
Iscozacin_L	12	50	266.4	147	0	0.488	0
Yanesha_L	11	24	77.5	302	0	3.139	0
Sanmatias_M	13	69	414	193	1	0.362	0.003
Yanachaga_H	15	89	433.7	204	13	0.403	0.028

To facilitate the identification of orchid bees in our videos, we caught orchid bees with entomological nets between 0900 and 1600 h for 3 days at each site. We always caught the orchid bees after we finished recording their pollination activity on the *Costus* flowers so that we did not interfere with their activity. We used pure chemical attractants eugenol, cineol, methyl salicylate, and benzyl acetate to attract them. We applied 0.10 mL of attractant to a cotton ball every half hour, and we used one cotton ball per attractant. We suspended the cotton balls at 1.50 m from the ground, 2 m apart among attractants. The captured individuals were euthanized and preserved with 70% ethanol for later identification in the Entomology Laboratory of the Universidad Nacional San Antonio Abad del Cusco, Peru, using a stereoscope (NOVEL NSZ‐608T). For the determination of the specimens, we used the terminology and taxonomic keys proposed by Dressler (1984), Bonilla‐Gomez and Nates‐Parra (1992), and Michener (2000). We tried to determine the orchid bee in our videos to species level when possible.

From our videos, we counted how many visits an inflorescence received and divided this number by the number of flowers present (visits per flower); then, we divided the visits per flower by the number of hours recorded to estimate the pollinator visitation rate per hour (hereafter visitation rate). We estimated the pollinator visitation rate at different taxonomic levels, such as family (Apidae and Trochilidae or pollinator group), and species or morphotypes. Moreover, we categorized bees into three groups based on their size, small (*Euglossa* spp), medium (*Eulaema* cf *mocsaryi*, *Eulaema* cf *polychroma*, *Eulaema* sp.), and big (*Eulaema* cf *bombiformis*, *Eufriesea* cf *ornata*), to estimate visitation rate by bee size. We decided to exclude the species *Aglae caerulea* from the big‐size bee group because it belongs to a different genus which is known to have ecological differences with species of the genus *Eulaema* (Roubik & Hanson, 2004) and we only recorded three visits of *A. caerulea* at Yanachaga_H site. We also constructed a standardized variable called pollinator morphotype abundance to compare pollinator assemblages among sites. This was calculated by dividing the visits per flower of each pollinator by the total number of pollinators visits at each site.

For the statistical analysis, we first compared the visitation rate of each pollinator group (bees separately from hummingbirds) among sites with a non‐parametric Kruskal–Wallis test, and a pairwise Wilcoxon post hoc test. Second, we compared the visitation rate of different orchid bee sizes among sites with a Kruskal–Wallis test and a pairwise Wilcoxon post hoc test. The visitation rate of big‐sized orchid bees was mostly zero in mid and low sites, then, for the Wilcoxon test, we indicated the alternative hypothesis of “less” for the group that had all or almost all zero values. Third, we compared the bee visitation rate by size within each site by applying the Kruskal–Wallis and the post hoc pairwise Wilcoxon tests. Fourth, we used the pollinator morphotype abundance to perform a Non‐metric Multidimensional Scaling (NMDS) using the *metaMDS* function in the package “vegan” (Oksanen et al., 2020). We applied a PERMANOVA and a multiple pairwise comparison test, to assess if pollinator assemblages differ among sites. Also, we ran a Simper analysis using the function *simper* from the package “vegan” to identify the pollinator morphotypes from each site that contributed to the dissimilarity with the other sites. Finally, from the bee sampling, we estimated orchid bee relative abundance in the different sites to evaluate if the orchid bee abundance changes along the elevational gradient. We applied a Kruskal–Wallis test and the post hoc Dunn's test (due to small and unequal sample size among the sites).

In addition, we carried out a survey of hummingbirds in the sites Iscozacin_L, Sanmatias_M, and Yanachaga_H during the 2020–2021 season. We conducted four walk transects of 1 km in each site and recorded the hummingbird species by observations (using binoculars) or songs. We looked up the bill length of the recorded hummingbird species in the literature (Hilty, 2002; Meyer et al., 1970; Schulenberg et al., 2010) to assess if they could reach the nectar of the flowers of *C. guanaiensis* var. *tarmicus* in the different sites. We considered hummingbird species that conducted a legitime visit to the flowers as having long enough bill length to reach the nectar of the flowers. We aimed to identify if there were other species with similar bill length in the sampled sites.

### 2.4 Floral phenotypes and pollinators size association

We determined whether floral size traits correlated with orchid bee body traits to evaluate if the different floral phenotypes were associated with the pollinator sizes. We chose the bee species with the highest visitation rate within each site to take the bee body measurements, assuming that the species most likely effecting the highest selection pressure on the floral traits would be the most frequent pollinator (Stebbins, 1970). We selected the species *Euglossa imperialis* in Iscozacin_L, *Euglossa intersecta* in Yanesha_L, *Eulaema mocsaryi* in Sanmatias_M, and *Eulaema bombiformis* in Yanachaga_H. We measured the bee thorax width and height, and the bee tongue (proboscis) length from 5 to 10 individuals of each orchid bee species. Using the mean of each variable, we applied Pearson's correlation between the following bee and flower traits, bee thorax width and the frontal corolla width, bee thorax height and the lateral corolla width, bee tongue length and corolla tube length (or the functional floral length that interacts with the bee's tongue), and bee tongue length and the distance between the anthers and the corolla base. We also conducted a bootstrap resampling from our empirical bee body measurements with replacement to pair each individual plant measurement with an individual bee measurement per site, thus, incorporating variation in each trait. We repeated the resampling for 1000 times and applied a Pearson's correlation to each originated dataset.

## 3 RESULTS

### 3.1 Floral phenotypes vary among sites

**FIGURE 2:**
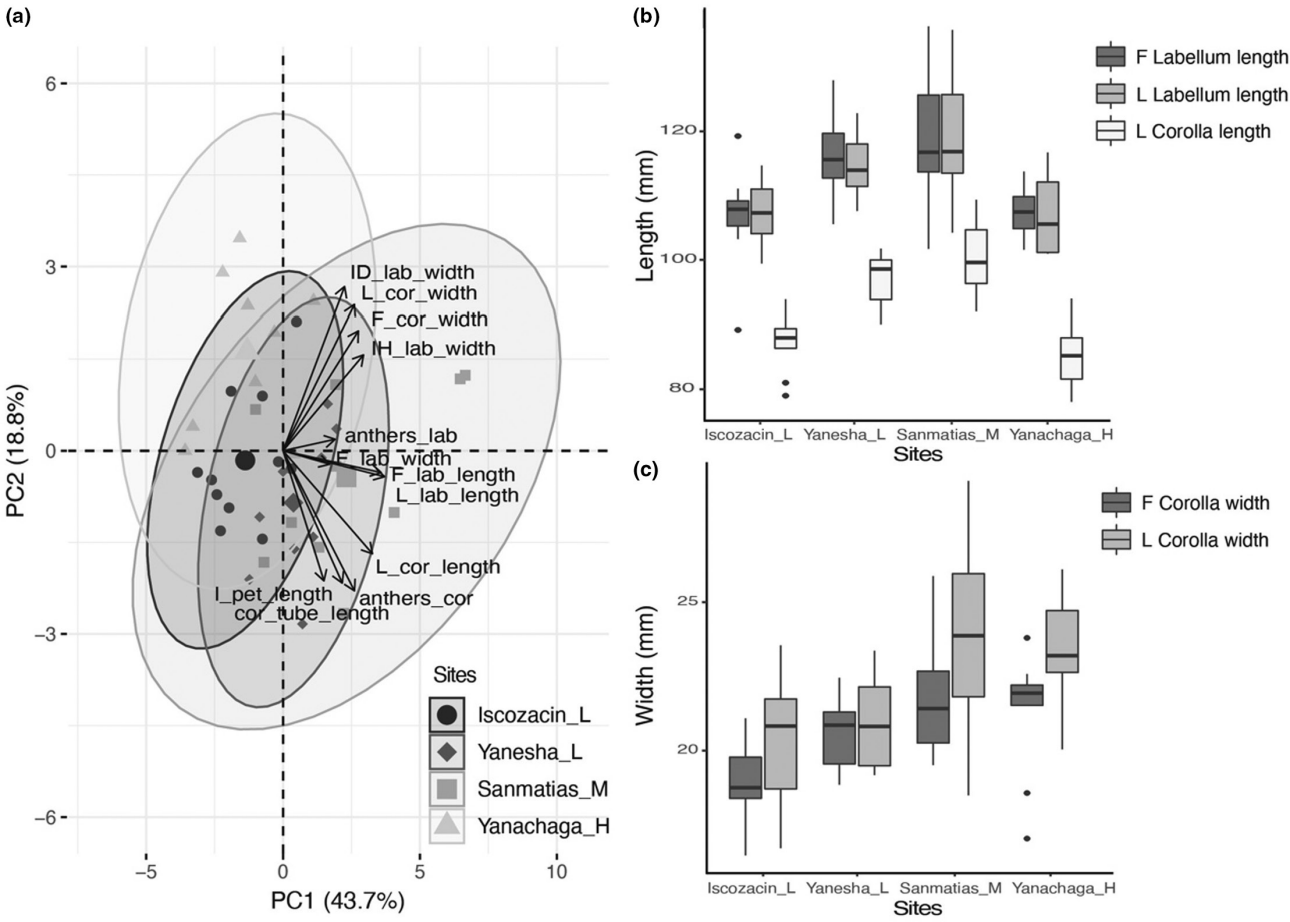
Flowers of *Costus guanaiensis* var. *tarmicus* vary in size among the sampled sites along the elevational gradient. (a) PCA plot using 12 floral traits measured from: frontal flower view, labellum length (F_lab_length), labellum width (F_lab_width), corolla width (F_cor_width); lateral flower view, labellum length (L_lab_length), corolla length (L_cor_length), corolla width (L_cor_width); internal flower view, petaloid stamen length above the stigma (I_pet_length), internal and horizontal labellum width (IH_lab_width), internal and diagonal labellum width (ID_lab_width), distance between the anthers and the labellum (anthers_lab), corolla tube length (cor_tube_length), distance between the anthers and the corolla base (anthers_cor). 95% confidence ellipses, ellipse centroid indicated by a larger symbol. (b) Boxplots of labellum and corolla length traits, and (c) corolla width traits from the four sampled sites.

We found that floral traits varied among sites. Flower size and nectar sugar concentration differed among two or more sites. The PCA using 12 floral trait measurements of size from four sites of *C. guanaiensis* var. *tarmicus* showed that PC1 captures 43.7% of the variation and PC2 captures 18.8%. Labellum and corolla length traits most correlated to PC1, whereas the lateral corolla width and the internal and diagonal labellum width most correlated to PC2. Grouping of flowers by site explains 34% of the floral size variation and this may be mainly driven by flower length traits (Figure 2a). The PERMANOVA test showed a significant difference in flower size among sites (*F* = 6.17, *R* ^2^ = .34, *p* = .001) and the multiple pairwise comparisons showed a significant difference in flower size between all site pairs (*p* < .05), except for Sanmatias_M—Yanesha_L and Yanachaga_H—Iscozacin_L.

The ANOVA test comparing the frontal measurements of the labellum length showed a significant difference among sites (*F* = 5.88, *p* = .002, Figure 2b) and Tukey's post hoc test showed a significant difference between the pairs Yanesha_L—Iscozacin_L, Sanmatias_M—Iscozacin_L and Sanmatias_M—Yanachaga_H (*p* < .05). The ANOVA test comparing the lateral measurements of the labellum length showed a significant difference among sites (*F* = 6.49, *p* = .001, Figure 2b) and Tukey's post hoc test showed a significant difference between the pairs Sanmatias_M—Iscozacin_L and Sanmatias_M—Yanachaga_H (*p* < .05). The ANOVA test comparing the lateral measurements of the corolla length showed a significant difference among sites (*F* = 21.89, *p* < .001, Figure 2b) and Tukey's post hoc test showed a significant difference between the pairs Yanesha_L—Iscozacin_L, Sanmatias_M—Iscozacin_L, Yanesha_L—Yanachaga_H and Sanmatias_M—Yanachaga_H (*p* < .05). Thus, we inferred that, Sanmatias_M has the largest flowers, Yanesha_L has intermediate flower length, and the other two sites have the shortest flowers. The ANOVA test comparing the frontal measurement of the corolla width showed a significant difference among sites (*F* = 5.41, *p* = .004, Figure 2c) and Tukey's post hoc test showed a significant difference between the pairs Sanmatias_M—Iscozacin_L and Sanmatias_M—Yanachaga_H (*p* < .05). The ANOVA test comparing the lateral measurement of the corolla width also showed a significant difference among sites (*F* = 7.04, *p* < .001, Figure 2c), the Tukey's post hoc test showed a significant difference between the pairs Sanmatias_M—Iscozacin_L, Yanachaga_H—Iscozacin_L, Sanmatias_M—Yanesha_L and Yanachaga_H—Yanesha_L. Thus, we inferred that, Sanmatias_M and Yanachaga_H have the widest flowers, whereas Iscozacin_L and Yanesha_L have the narrowest ones.

**FIGURE 3:**
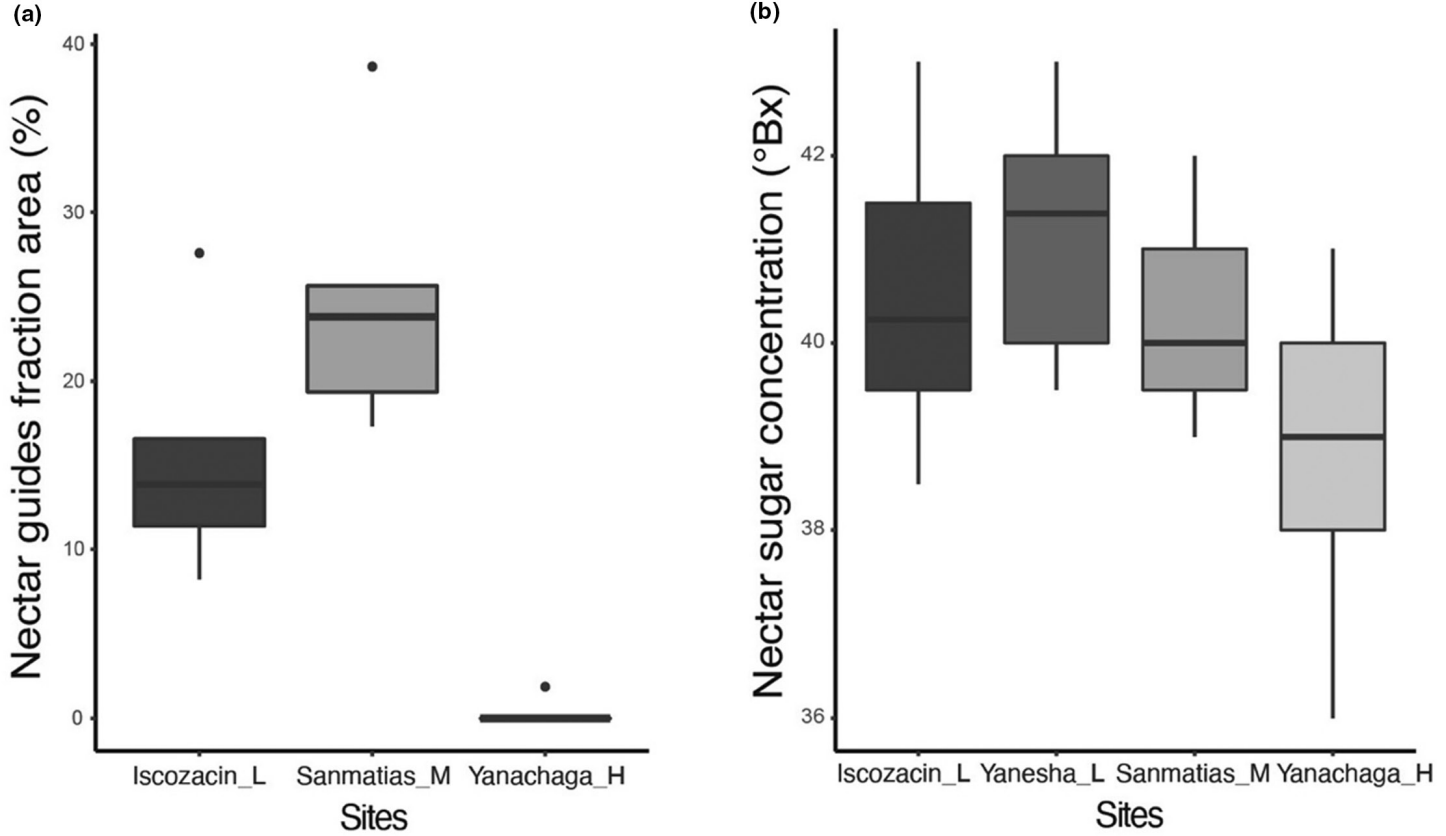
Flowers of *Costus guanaiensis* var. *tarmicus* vary in color and nectar sugar concentration among the sampled sites along the elevational gradient. (a) Boxplot of nectar guides fraction area on the labellum from three sites. (b) Boxplot of nectar sugar concentration in degrees Brix (°Bx) from four sites.

We found variation in nectar guides fraction area among sites (Kruskal–Wallis, *χ* ^2^ = 11.02, df = 2, *p* = .004). Dunn's test revealed that Yanachaga_H was significantly different than Sanmatias_M (*p* = .002) and Iscozacin_L (*p* = .05). But the latter two did not differ between them. Thus, the nectar guides fraction area was the smallest in Yanachaga_H (*x̄* = 0.37%, SD ± 0.83, Figure 3a). Similarly, we found variation in nectar sugar concentration among sites (ANOVA, *F* = 6.47, *p* < .001). The Tukey's post hoc test showed that Yanachaga_H was significantly different from the other three sites (*p* < .05). Thus, Yanachaga_H had the lowest nectar sugar concentration (*x̄* = 38.9°B, SD ± 1.4), whereas the other three sites had similar levels of nectar sugar concentration (Figure 3b).

### 3.2 Pollinator assemblages vary among sites

We video‐recorded a total of 1192 h of pollinator activity on the flowers of *C. guanaiensis* var. *tarmicus*. The flowers were visited by bees and hummingbirds (Table 1). We recorded orchid bees from four genera pollinating the flowers. By contrasting the video images with our orchid bee collection, we identified the following species pollinating the flowers, *Euglossa* cf *imperialis*, *Euglossa intersecta*, *Euglossa* cf *magnipes*, *Euglossa* cf *mixta*, *Eulaema* cf *cingulata*, *Eulaema* cf *mocsaryi*, *Eulaema* cf *polychroma*, *Aglae caerulea*. There were some individuals that we could not identify at the species level, which we recorded as *Euglossa* sp., *Eulaema* sp., and a morphotype composed of *Eulaema bombiformis* and *Eufriesea ornata*. In addition, there were a few legitimate visits by a bumble bee (*Bombus* sp.) and a stingless bee (*Melipona* sp.). Also, there were some legitimate visits from hummingbirds, mainly from the green hermit *Phaethornis guy*, and only one visit from the great‐billed hermit *Phaethornis malaris*.

We caught orchid bees from the genera *Euglossa*, *Eulaema*, *Eufriesea*, and *Exaerete*. We found a significant difference in orchid bees' relative abundance among the sites (Kruskal–Wallis, *χ* ^2^ = 8.48, df = 3, *p* = .047), and Dunn's test revealed that Yanachaga_H had a lower bee abundance than Yanesha_L (*p* = .026). We recorded 21 species of hummingbirds in our survey (9 in Iscozacin_L, 9 in Sanmatias_M, and 11 in Yanachaga_H, see Appendix 3 for the complete list of species recorded in each site). The species with the longest bill were *Phaethornis guy* (40 mm, Schulenberg et al., 2010) in Yanachaga_H, and *Phaethornis malaris* (45 mm, Meyer et al., 1970) in Sanmatias_M and Iscozacin_L, corresponding to the same species that visited the flowers of *C. guanaiensis* var. *tarmicus*. The species with the next longest bill reached 34 mm.

Orchid bee visitation rate differed among sites (Kruskal–Wallis, *χ* ^2^ = 28.224, df = 3, *p* < .001), with Yanesha_L having the highest bee visitation rate (*x̄* = 3.13, SD ± 3.18, pairwise Wilcoxon tests between Yanesha_L and each of the other sites, *p* < .001). The bee visitation rate among the other three sites did not differ. Similarly, we found a significant difference in hummingbird visitation rate among sites (Kruskal–Wallis, *χ* ^2^ = 10.687, df = 3, *p* = .01); Yanachaga_H had the highest hummingbird visitation rate (*x̄* = 0.03, SD ± 0.15; Wilcoxon test between Yanachaga_H and each of the other sites, *p* ≤ .05). However, the hummingbird visitation rate is only approximately 7% of the bee visitation rate in Yanachaga_H (Table 1).

**FIGURE 4:**
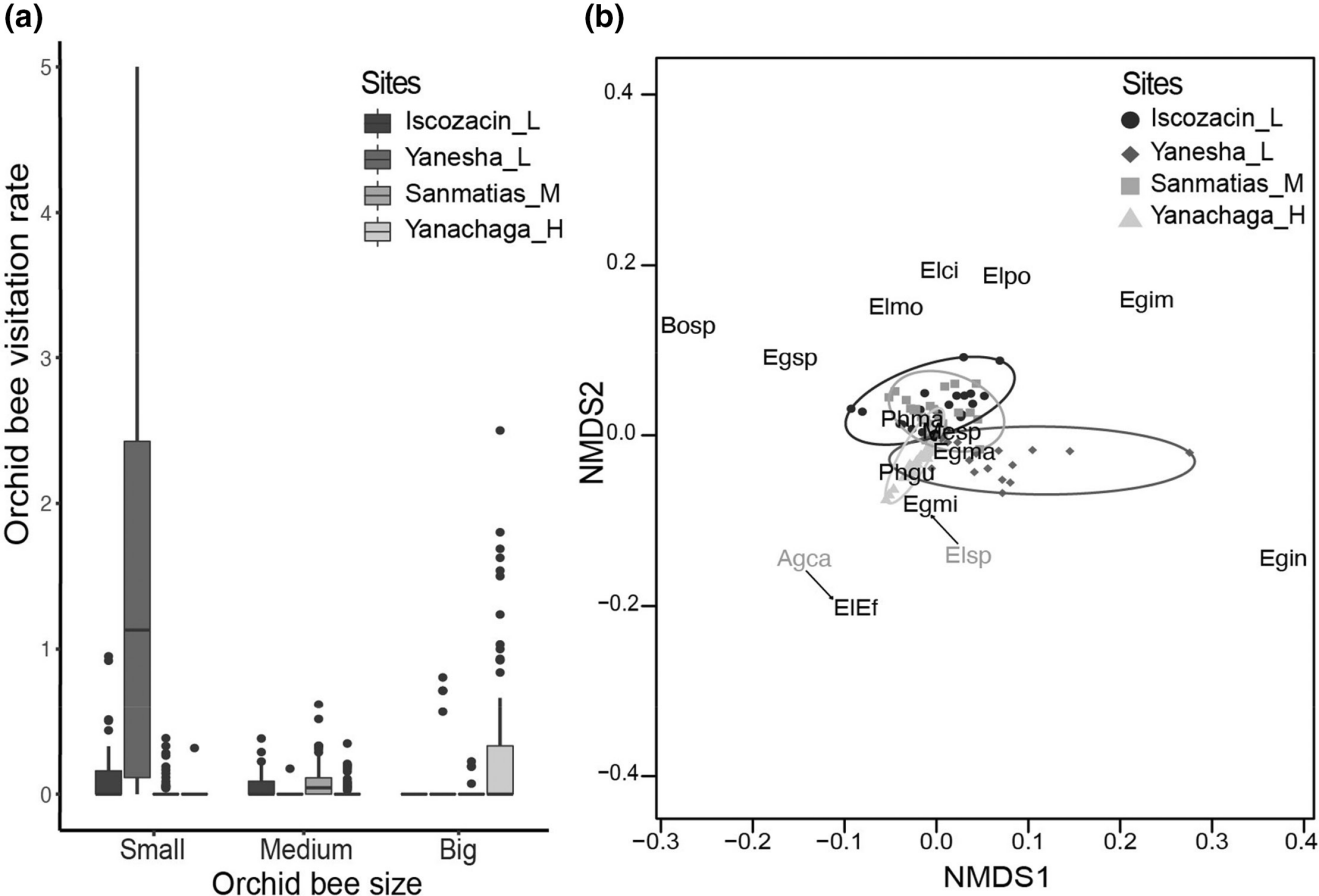
Pollinator assemblages of *Costus guanaiensis* var. *tarmicus* vary in orchid bee size and assemblage composition among the sampled sites along the elevational gradient. (a) Boxplot of visitation rate of orchid bees categorized by size as small, medium, and big from the sampled sites. (b) Nonmetric Multidimensional Scaling plot of pollinator assemblages composed by bees and hummingbirds from the sampled sites. 95% confidence ellipses. Arrows indicate an overlap between two pollinators in the multidimensional space; both pollinators occupy the same space at the end of arrow. Orchid bee size grouping and abbreviations: Small orchid bees include morphotypes *Euglossa* cf *imperialis* (Egim), *Euglossa intersecta* (Egin), *Euglossa* cf *magnipes* (Egma), *Euglossa* cf *mixta* (Egmi), *Euglossa* sp. (Egsp), medium orchid bees include morphotypes *Eulaema* cf *cingulata* (Elci), *Eulaema* cf *mocsaryi* (Elmo), *Eulaema* cf *polychroma* (Elpo), *Eulaema* sp. (Elsp), big orchid bees include morphotypes *Aglae caerulea* (Agca), *Eulaema bombiformis—Eufriesea ornata* (EIEf). Other bees: *Bombus* sp. (Bosp), *Melipona* sp. (Mesp). Hummingbirds: *Phaethornis guy* (Phgu), *Phaethornis malaris* (Phma).

We compared the visitation rates of small, medium, and big‐sized orchid bees among sites (Figure 4a). We found a significant difference among sites for small‐sized bees (Kruskal–Wallis, *χ* ^2^ = 87.746, df = 3, *p* < .001), and the pairwise Wilcoxon test showed a significant difference between all site pairs (*p* < .01), resulting in a decrease of visitation rate of small‐size bees in the following order, Yanesha_L, Iscozacin_L, Sanmatias_M, and Yanachaga_H. There was also a significant difference in visitation rate for medium‐sized bees (Kruskal–Wallis, *χ* ^2^ = 29.304, df = 3, *p* < .001), the pairwise Wilcoxon test showed that the visitation rate of medium‐sized bees is higher in Iscozacin_L and Sanmatias_M than in the other two sites (*p* ≤ .01). Finally, there was a significant difference in visitation rate for big‐sized bees (Kruskal–Wallis, *χ* ^2^ = 34.497, df = 3, *p* < .001). The visitation rate of big‐sized bees was higher in Yanachaga_H (pairwise Wilcoxon tests comparing Yanachaga_H with each of the other sites *p* < .05), and decreases in the following order, Yanesha_L, Sanmatias_M, and Iscozacin_L. In addition, comparing the visitation rate by bee size within sites we found that in both low sites, Iscozacin_L and Yanesha_L, small‐sized bees had the highest visitation rate; in the mid site, Sanmatias_M, medium‐sized bees had the highest visitation rate, and in the high site, Yanachaga_H, big‐sized bees had the highest visitation rate (Table 2). Thus, the highest site is mainly pollinated by big‐sized bees, the mid site by medium‐sized bees, and the low sites by small‐sized bees.

**TABLE 2:** *p*‐Values from the pairwise Wilcoxon tests comparing orchid bee visitation rate by size within each sampling site in our elevational gradient.

Iscozacin_L			Yanesha_L		
	Small	Medium		Small	Medium
Medium	.33	–	Medium	<.001	‐
Big	<.001	<.001	Big	<.001	.07

Sanmatias_M			Yanachaga_H		
	Small	Medium		Small	Medium
Medium	<.001	‐	Medium	<.001	‐
Big	.01	<.001	Big	<.001	<.001

Finally, the PERMANOVA test showed a significant difference between the sites based on the NMDS projection of the pollinator morphotypes' abundance (*F* = 7, *R* ^2^ = .08, *p* = .001, Figure 4b). The multiple pairwise comparisons showed a significant difference between all the pairs (*p* < .01). The Simper analysis showed that *Euglossa* cf *imperialis* differentiated the Iscozacin_L pollinator assemblage from the rest (contributing between 20.5% and 30.1% to the dissimilarity), *Euglossa intersecta* in Yanesha_L (contributing between 56.3% and 64.3%), *Eulaema* cf *mocsary* in Sanmatias_M (contributing between 18.1% and 38.5%), and the compound morphotype of *Eulaema bombiformis* and *Eufriesea ornata* in Yanachaga_H (contributing between 16% and 30.8%) Thus, the pollinator assemblages differed among sites by the presence of hummingbirds and because there were different orchid bees assemblages at each site.

### 3.3 Floral phenotype and pollinator size association

**FIGURE 5:**
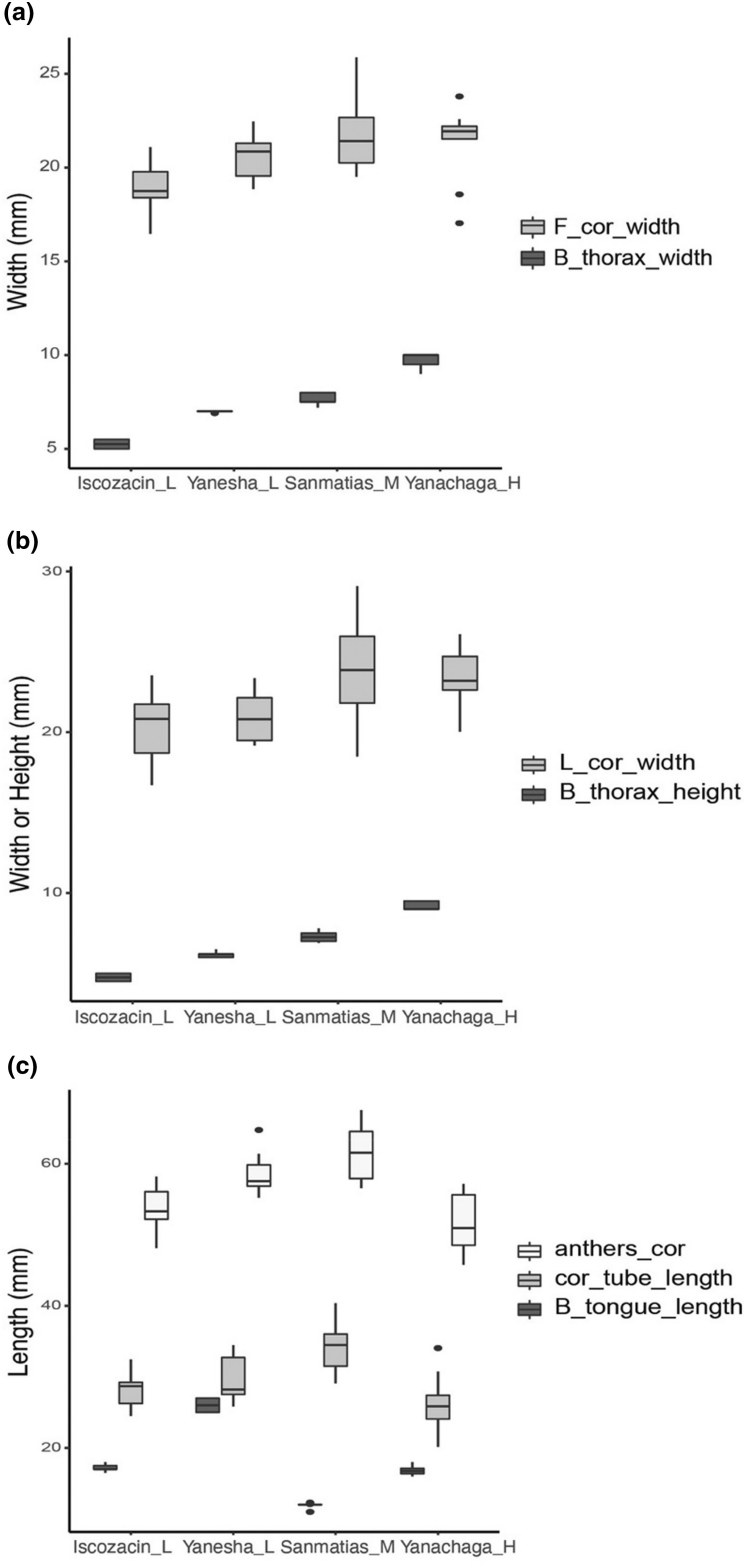
The association between the bee body traits of the species with the highest visitation rate per site and the flower size traits. (a) A positive correlation between the bee thorax width and the frontal corolla width. (b) A positive correlation between the bee thorax height and the lateral corolla width. (c) No correlation between the bee tongue length and the corolla tube length, and between the bee tongue length and the distance between the anthers and the corolla base.

We tested if floral traits and bee traits covaried using Pearson's correlation test. We found a non‐significant correlation between our site‐averaged variables, as expected with four sites. The correlation between the bee body size traits and the floral width traits showed positive correlation coefficients higher than .75 (Figure 5a,b), whereas the correlation between the tongue length and the corolla tube length, and the tongue length and the distance between the anthers and the corolla base showed a negative correlation coefficient lower than .4 (Figure 5c). After bootstrap resampling and repeated correlation, we found a positive significant correlation between bee thorax width and the frontal corolla width (1000 out of 1000 repetitions with a *p* < .05, .34 < coef < .5), and between the bee thorax height and the lateral corolla width (1000 out of 1000 repetitions with a *p* < .05, .42 < coef < .57). We did not find a significant correlation between the bee tongue length and the corolla tube length (61 out of 1000 repetitions with a *p* < .05), nor between the bee tongue length and the distance between the anthers and the corolla base (0 repetitions out of 1000 with a *p* < .05).

## 4 DISCUSSION

Our results showed clear variation in floral traits and pollinator assemblages in *C. guanaiensis* var. *tarmicus* across a steep elevational gradient from the Amazon to the foothills of the Andes in Peru. We also found an association between floral traits and bee body traits contributing to the mechanical flower‐pollinator fit. The substantial divergence in both floral traits and pollinator assemblages suggests the establishment of the necessary preconditions for pollinator‐driven divergence. Our findings highlight the importance of assessing floral traits and pollinator assemblages' geographic variation in the Neotropics by allowing us to learn from a new plant study system and novel composition of pollinator assemblages that are not found in temperate areas (orchid bees and hermit hummingbirds). Below we examine our results in light of two hypotheses for how variation is structured: that it could reflect local adaptation to different assemblages of the same pollinator functional group (bees) or that it could reflect the early stages of an evolutionary shift to a new pollinator functional group (hummingbirds).

### 4.1 Is there evidence for the local bee adaptation hypothesis?

The local bee adaptation hypothesis predicts that the pollinator assemblages of *C. guanaiensis* var. *tarmicus* comprise only bees in all the different elevational sites and the floral traits vary among sites. We found support for this hypothesis in the dissimilarity of flower sizes and in the presence of bees as part of all pollinator assemblages along the elevational gradient. Also, the association found between floral traits and bee body traits further supports this hypothesis. The corolla size (frontal and lateral corolla width) changes with the bee thorax size (width and height) of the most frequent pollinator, with both traits increasing along the elevational gradient. The variation in corolla size may promote a better fit between flowers and bees. Our finding coincides with the pattern found by previous studies that higher elevation populations have wider flowers and are pollinated by bigger bees (bumble bees) compared to lower populations (*Polemonium viscosum* in the Rocky Mountains by Galen, 1996; and *Campanula rotundifolia* in the Norwegian mountains by Maad et al., 2013). Previous studies found an association between the flower length and the pollinator mouth part length (Maad et al., 2013; Nagano et al., 2014), which we did not find in our study. Perhaps the flower length is not a trait upon which orchid bees’ tongue length exerts a selective pressure, given that the bees crawl inside the flowers of *Costus* to feed on the nectar. Studies about the evolution of nectar flowers for orchid bees suggested that long flowers for orchid bees might have evolved via competition among sympatric species for attracting traplining pollinators that would include them in their feeding routes (Borrell, 2005; Garrison & Gass, 1999; Rathcke, 1992), and not via directional selection exerted by specialized pollinators (Darwin, 1862), such as those with extreme trait adaptations. In addition, the local bee adaptation hypothesis stated that if there is a low bee visitation rate in the highest site, we should see an exaggeration of bee attraction traits there. Our results did not support this part of the hypothesis; we neither found a lower bee visitation rate nor exaggerated bee attraction traits in the highest site. This finding contrasts with the study of Dellinger et al. (2021), who found a decrease in bee visitation rate with elevation in Merianieae species, but their high elevation sites were located beyond 1800 m a.s.l. Altogether, we found evidence that supports the adaptation of *C. guanaiensis var. tarmicus* flowers to the local bee fauna along the studied elevational gradient.

### 4.2 Is there evidence for the hummingbird pollination shift hypothesis?

The pollinator shift hypothesis predicts that the pollinator assemblage of *C. guanaiensis* var. *tarmicus* at the highest elevation site would comprise bees and hummingbirds, but with the lowest bee visitation rate and the highest hummingbird visitation rate compared to lower sites. The floral traits of the highest site would include traits that deter bees and/or attract‐fit hummingbirds. Our main finding supporting this hypothesis is the presence of hummingbirds of the species *Phaethornis guy* as part of the pollinator assemblage of only the highest site. Even though we recorded a hummingbird species with a similar bill size in the lower sites (*P. malaris*), we did not record hummingbirds feeding on the *C. guanaiensis* var. *tarmicus* flowers there. The feeding of *P. guy* on *C. guanaiensis* var. *tarmicus* flowers in the highest site could be due to two non‐mutually exclusive factors, the presence of hummingbird fitting or attracting floral traits in *C. guanaiensis* var. *tarmicus*, and a decrease in hummingbird floral resources in the area (Biesmeijer et al., 2006; Maglianesi et al., 2015; Ornelas et al., 2007; Smith et al., 1995). Regarding hummingbird fitting floral traits, the short corolla in the flowers of the highest site should make those flowers accessible to the hummingbirds. In addition, the level of sugar concentration in the nectar of the highest site (*x̄* = 38.9° Brix, SD ± 1.4) might work as a hummingbird attracting floral trait. This is because the sugar level in the highest site is higher than the sugar level found in many hummingbird‐visited flowers (Baker, 1975; Bolten & Feinsinger, 1978; Chalcoff et al., 2006; McDade & Weeks, 2004; Rodríguez‐Flores & Stiles, 2005), including hummingbird‐pollinated *Costus* species (Rodríguez‐Flores & Stiles, 2005; Sytsma & Pippen, 1985). We initially thought that the level of sugar concentration in the nectar found in the highest site might be related to bee deterrence since it is lower than the one found in the lower sites. However, the sugar level found in the nectar of the highest site is within the range of sugar level of most flowers foraged by euglossine bees (30%–40%, Roubik et al., 1995). Regarding the availability of other hummingbird floral resources, unfortunately, we did not account for other hummingbird floral resources in our studied sites to analyze if there are lesser available resources in the highest site compared to lower sites. Maglianesi et al. (2015) showed that if there is a decrease in hummingbird floral resources, hummingbirds can show a less specialized diet feeding on a larger number of plant species. Moreover, Stiles (1978) reported hermit hummingbirds feeding on a bee‐pollinated *Costus* species (*C. malortieanus*) in Costa Rica at the end of the rainy season (November–December) which matches the low flowering point of hummingbird foodplants during the year.

Another finding supporting the pollination shift hypothesis is the reduced nectar guides fraction area in the highest site compared to lower sites. Schemske and Bradshaw (1999) found that the removal of nectar guides on the *Mimulus* flowers lowered the bee visitation rate. In our study, we did not find a decrease in bee visitation rate, perhaps the nectar guides area must be completely absent to influence bee visitation. Overall, we found some evidence supporting the pollinator shift hypothesis. Although the association between the corolla size and the bee thorax size of the most frequent pollinator could contradict this hypothesis, the two scenarios might not be mutually exclusive.

### 4.3 Floral traits and pollinator assemblages differed between the lowest sites

Flower length differed between the two lowest sites, Iscozacin_L has shorter flowers than Yanesha_L. Moreover, we also found differences in pollinator assemblages and pollinator visitation rates between them. These findings suggest that other factors beyond the elevation can promote variation in floral traits and pollinator assemblages.

Furthermore, the Yanesha_L bee's visitation rate was the highest among all the sites. This result might be driven by differences in the level of habitat degradation among sites. Yanesha_L is in the middle of protected areas, whereas Iscozacin_L is outside of a protected area surrounding a small town. The other sites, Sanmatias_M and Yanachaga_H, are on the border of protected areas with roads crossing these sites (Figure 1). Previous studies showed that orchid bee abundance and richness are higher in well‐preserved areas compared to disturbed areas (Storck‐Tonon & Peres, 2017). Our findings contribute evidence to the importance of habitat conservation for orchid bees and their role as pollinators, considering that, in average, the bee visitation rate inside a protected area was 7 times higher compared to unprotected areas. This striking difference may also affect the reproductive success of our native studied plant species *C. guanaiensis* var. *tarmicus*.

### 4.4 Correlation or causation? Caveats about the association of floral traits and pollinator assemblages

Some caveats about correlation versus causation for the association of floral traits and pollinator assemblages merit discussion. First, we do not know the mechanism behind the correlation we found between the corolla size and the bee thorax size. The flower size may adapt to the available pollinators, or the flower size may vary due to pollination‐unrelated factors and the pollinators preferentially feed on the right size of flowers for them (Nagano et al., 2014). If the latter mechanism was true, we might see a wider variation of the floral trait in a site with a wide range of bee sizes visiting the flowers. Our best option to observe this would be the Yanesha_L low site, where we observed the widest range of bee sizes visiting the flowers. However, the floral size range there does not surpass the floral size range of other sites with a narrower range of bee sizes visiting the flowers. The lack of the proposed scenario suggests that the available pollinator assemblage is what might influence the variation in the floral size of *C. guanaiensis* var. *tarmicus*. A proper analysis of the mechanism would involve conducting an experiment exposing different flower sizes to bees of different sizes and testing whether by feeding on a flower size that correlates with their own size the pollination efficiency is higher than when there is a flower‐pollinator trait mismatch. Second, our goal was to relate the observed floral phenotypes with the pollinator assemblages, not to test for floral local adaptation to pollinators. A proper test of local floral adaptation would be to conduct a reciprocal transplant experiment with cloned plants from different elevations to test whether clones would attract a pollinator assemblage similar to its native pollinator assemblage in every location, achieving similar levels of pollination efficiency as in their native sites and whether clones from native populations would have higher pollinator visitation rate (or any other measure of reproductive success) than the introduced populations (Newman et al., 2015; Streisfeld & Kohn, 2007). The logistics for an experiment of this nature are tremendous in places like Peru, which lack the infrastructure to grow *Costus* clones for experiment replicates in most of the research sites we visited, and the unpaved roads make preclude transporting clones between distant sites. Finally, it is difficult to understand which floral traits are targets of pollinator‐mediated selection with an assessment of floral phenotype and pollinator assemblage correlation. Conducting studies hybridizing populations or species to increase phenotypic variance (Schemske & Bradshaw, 1999) or artificially manipulating single and combined floral traits and exposing them to different pollinator groups would help to elucidate any selected traits, as well as specific floral traits that function as anti‐bee and/or pro‐bird traits as it has been done in temperate plant systems (Castellanos et al., 2004; Gegear et al., 2017; Salas‐Arcos et al., 2019; Zung et al., 2015, but see Bergamo et al., 2016, for a study of tropical flowers).

## 5 CONCLUSIONS

We found substantial divergence in floral traits of a Neotropical *Costus* species and its pollinator assemblage across a steep elevational gradient spanning the Amazonian lowlands to the eastern foothills of the Central Andes, establishing the necessary preconditions for pollinator‐driven divergence. Taking together the results from floral traits and pollinator assemblages variation suggest that the populations of *C. guanaiensis* var. *tarmicus* are adapted to the local bee fauna along the studied elevational gradient, but we cannot rule out the possibility of the beginning of a bee‐to‐hummingbird pollination shift in the highest studied site.

## AUTHOR CONTRIBUTIONS

**Rossana Maguiña‐Conde:** Conceptualization (equal); data curation (lead); formal analysis (lead); funding acquisition (lead); investigation (lead); methodology (lead); project administration (lead); resources (lead); software (lead); supervision (lead); validation (lead); visualization (lead); writing – original draft (lead); writing – review and editing (lead). **Dorali Zuñiga‐Rivas:** Data curation (supporting); investigation (supporting); methodology (supporting); resources (supporting); writing – review and editing (supporting). **Kathleen M. Kay:** Conceptualization (equal); funding acquisition (supporting); investigation (supporting); methodology (supporting); project administration (supporting); resources (supporting); supervision (supporting); writing – original draft (supporting); writing – review and editing (supporting).

## CONFLICT OF INTEREST STATEMENT

The authors declare no competing interests.

## Figures and Tables

**Figure:**
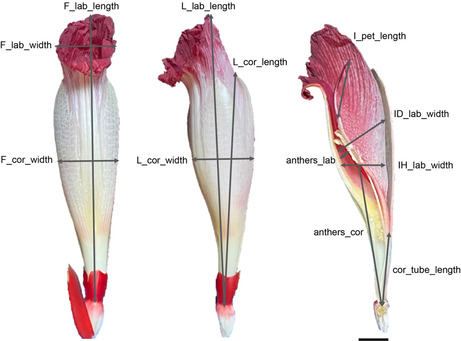
Diagram of floral traits reference for *C. guanaiensis* var. *tarmicus* flowers. From left to right, frontal view, lateral view and internal view of flower. anthers_cor, the distance between the anthers and the corolla base; anthers_lab, the distance between the anthers and the labellum; cor_tube_length, corolla tube length; F_cor_width, corolla width; F_lab_length, frontal view, labellum length; F_lab_width, labellum width; I_pet_length, Internal view, petaloid stamen length; ID_lab_width, internal and diagonal labellum width; IH_lab_width, internal and horizontal labellum width; L_cor_length, corolla length; L_cor_width, corolla width; L_lab_length, lateral view, labellum length. Scale bar is 10 mm.

**Figure:**
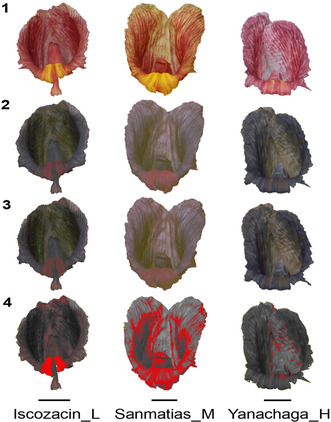
Flowers of *C. guanaiensis* var. *tarmicus* vary in the size of the nectar fraction area in the labellum among three sampled sites (columns) along the elevational gradient. (1) Pictures taken with a human visible‐spectrum‐pass filter under natural day light. (2) Presentation image for human vision based on a honey bee cone catch image. We assumed that the UV reflectance colors correspond to the nectar guides of the flower, and appear as dark pink‐purple colors. (3) Image B converted to 8‐bit color type image with 256 colors. (4) The 8‐bit grayscale type image width the threshold levels adjusted to identify the pixels corresponding to the dark pink‐purple nectar guides. Scale bars are 10 mm.

**Figure:**
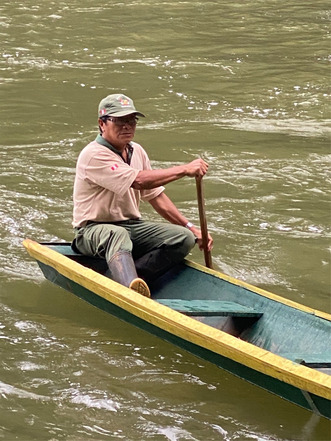
Avelino Sebastián Espinoza

## Data Availability

All data analyzed in the manuscript will be made accessible through the public Dryad repository. https://datadryad.org/stash/share/_Y74fAaqB4mNN4rWugtTtosIUCY4aaBQw50AR1tf6Os.

## APPENDIX 3

### List of hummingbirds' species from our survey and their bill length

**Table:**

Species name	Bill length (mm)	Iscozacin_L	Sanmatias_M	Yanachaga_H
*Adelomya melanogenys*	15^a^			x
*Anthracothorax nigricollis*	25^a^	x		
*Campylopterus largipennis*	30^a^	x	x	
*Chaetocercus mulsant*	17^a^			x
*Chlorostilbon mellisugus*	15^a^	x	x	x
*Doryfera ludovicae*	34^a^			x
*Florisuga mellivora*	20^a^	x		x
*Glaucis hirsutus*	30^a^	x	x	
*Heliodoxa aurescens*	20^a^			x
*Metallura tyrianthina*	12^a^			x
*Ocreatus underwoodii*	16^a^			x
*Phaethornis guy*	40^a^			x
*Phaethornis hispidus*	34^a^	x		
*Phaethornis malaris*	45^b^	x	x	
*Phaethornis ruber*	23^a^	x	x	
*Phaethornis stuarti*	23^a^		x	
*Phlogophilus harterti*	15^a^			x
*Talaphorus chlorocercus*	17^c^		x	
*Thalurania furcata*	20^a^	x	x	x
*Threnetes leucurus*	32^a^		x	
Total		9	9	11

^a^ a Schulenberg et al. (2010).

^b^ b Meyer et al. (1970).

^c^ c Hilty (2002).
